# Quercetin Inhibits Fibroblast Activation and Kidney Fibrosis Involving the Suppression of Mammalian Target of Rapamycin and β-catenin Signaling

**DOI:** 10.1038/srep23968

**Published:** 2016-04-07

**Authors:** Jiafa Ren, Jianzhong Li, Xin Liu, Ye Feng, Yuan Gui, Junwei Yang, Weichun He, Chunsun Dai

**Affiliations:** 1 Center for Kidney Diseases, 2nd Affiliated Hospital, Nanjing Medical University, 262 North Zhongshan Road, Nanjing, Jiangsu, China; 2 State Key Laboratory of Reproductive Medicine, Institute of Toxicology, Nanjing Medical University, Nanjing, Jiangsu, China

## Abstract

Quercetin, a flavonoid found in a wide variety of plants and presented in human diet, displays promising potential in preventing kidney fibroblast activation. However, whether quercetin can ameliorate kidney fibrosis in mice with obstructive nephropathy and the underlying mechanisms remain to be further elucidated. In this study, we found that administration of quercetin could largely ameliorate kidney interstitial fibrosis and macrophage accumulation in the kidneys with obstructive nephropathy. MTORC1, mTORC2, β-catenin as well as Smad signaling were activated in the obstructive kidneys, whereas quercetin could markedly reduce their abundance except Smad3 phosphorylation. In cultured NRK-49F cells, quercetin could inhibit α-SMA and fibronectin (FN) expression induced by TGFβ1 treatment. MTORC1, mTORC2, β-catenin and Smad signaling pathways were stimulated by TGFβ1 at a time dependent manner. Similar to those findings in the obstructive kidneys, mTORC1, mTORC2 and β-catenin, but not Smad signaling pathways were remarkably blocked by quercetin treatment. Together, these results suggest that quercetin inhibits fibroblast activation and kidney fibrosis involving a combined inhibition of mTOR and β-catenin signaling transduction, which may act as a therapeutic candidate for patients with chronic kidney diseases.

Kidney interstitial deposition of extracellular matrix (ECM), one of the pathological features for chronic kidney diseases (CKD), may lead to progressive kidney fibrosis and end stage of renal disease (ESRD) in patients^1^,^2^. Once activated, myofibroblasts can produce a large amount of extracellular matrix (ECM) to initiate and promote kidney fibrosis^3^. Although many cell types in the kidney, including fibroblast, tubular epithelial cell, pericyte as well as endothelial cell contribute to myofibroblast accumulation, fibroblast is thought to be the major source for myofibroblast in the fibrotic kidney tissue^1^,^4^. Blockade of fibroblast activation should shed light on prohibiting kidney fibrosis and halting the progression of CKD.

During the past decades, the mechanisms for kidney fibrosis have been deeply investigated, but few therapeutic strategies are available to efficiently halt the progression of interstitial fibrosis and chronic kidney diseases^5^,^6^. Recently, Qin *et al*. tested the anti-fibrotic activities of 21 compounds isolated from plants used in Chinese medicine and revealed that quercetin, a flavonoid found in a wide variety of plants and presented in human diet, displays promising potential in preventing kidney fibroblast activation^7^. Quercetin has wide range of biological activities including anti-oxidation, anti-inflammatory and anti-tumor^8^,^9^,^10^,^11^. Regarding the beneficial effect for quercetin in protecting against fibrosis in the liver, lung and skin^12^,^13^,^14^,^15^,^16^,^17^, it is highly possible that quercetin may be effective in prohibiting kidney fibroblast activation as well as kidney fibrosis in mice with obstructive nephropathy.

Accumulative evidences demonstrated that transforming growth factor-β1 (TGFβ1), a central mediator of fibrogenesis, may activate a panel of intracellular signaling pathways including Smad, β-catenin and mTOR^1^,^18^,^19^,^20^,^21^, and inhibiting such signaling pathways is protective against fibroblast activation and kidney fibrosis. Although a large amount of evidence supports that Smad signaling plays a crucial role in governing TGFβ1–induced fibrosis and SIS3, a Smad3 inhibitor, delays the early development of diabetic nephropathy^22^, several other signaling pathways also contribute to TGFβ1-induced fibroblast activation and kidney fibrosis. Our and the other studies reported that targeting mTORC1 signaling with rapamycin reduces kidney fibroblast activation and fibrosis^21^,^23^. Specific deletion of Rictor, a major component of mTORC2, also diminishes TGFβ1-induced fibroblast activation and kidney fibrosis^24^. Additionally, TGFβ1 treatment may also activate Wnt/β-catenin signaling in lung fibroblasts, whereas a lack of β-catenin attenuates the ability of TGFβ1 to promote fibroblast proliferation. A peptidomimetic small molecule ICG-001, which specifically disrupts β-catenin-mediated gene transcription, ameliorates UUO nephropathy in mice^25^. It is of note that quercetin may inhibit mTOR and β-catenin signaling in cancer cells^26^,^27^,^28^,^29^. Given the critical role for mTOR and β-catenin signaling in mediating TGFβ1-induced fibroblast activation and kidney fibrosis, we hypothesized that quercetin may suppress fibroblast activation and kidney fibrosis involving the suppression of these signaling pathways.

In this study, we found that administration of quercetin markedly retarded the progression of renal fibrosis, macrophage accumulation as well as the inflammatory cytokines expression in the obstructive kidneys in mice. MTOR and β-catenin signaling pathways but not Smad signaling were largely diminished in quercetin-treated kidneys. In cultured rat kidney interstitial fibroblast cells, quercetin effectively blocked TGFβ1-induced fibroblast activation. Similar to the *in vivo* studies, the activation of mTOR and β-catenin signaling pathways stimulated by TGFβ1 treatment were blocked by quercetin, while Smad3 phosphorylation remained unchanged.

## Results

### Quercetin ameliorates obstructive nephropathy in mice

**Figure 1:**
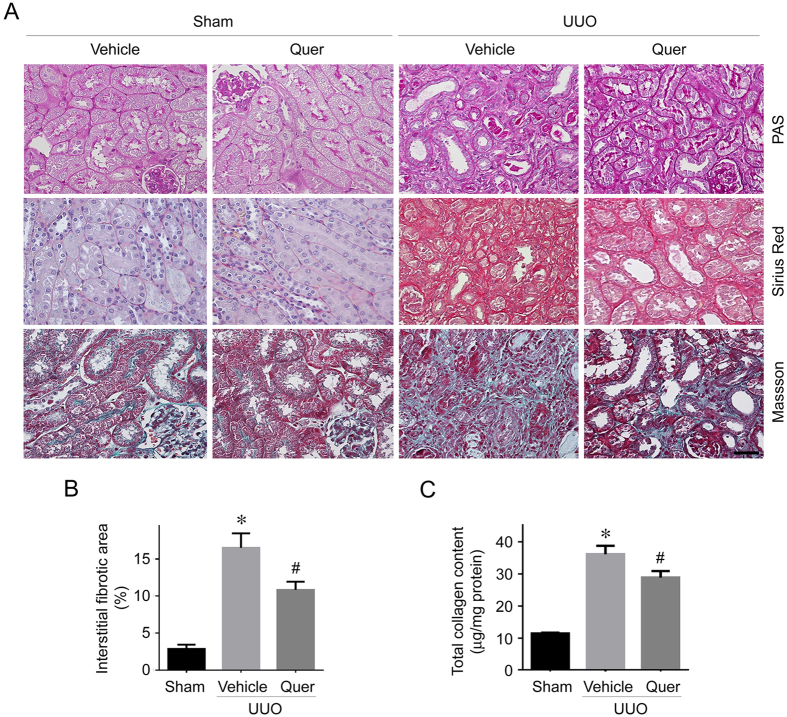
Quercetin ameliorates UUO nephropathy in mice. Male CD-1 mice received UUO operation were treated with quercetin (25 mg/kg.day) and sacrificed at 2 weeks after surgery. (**A**) Representative micrographs for periodic acid–Schiff (PAS), Masson and Sirius red staining in the kidneys from different groups as indicated. Scale bar = 50 μm. (**B**) Graphic presentation showing the percentage of fibrotic area in the kidneys from different groups as indicated. *P < 0.05 compared with Sham kidneys (*n* = 3–7); ^#^*P* < 0.05 compared with UUO kidneys with vehicle treatment (*n* = 7). (**C**) Graphic presentation showing total collagen content in the kidneys from different groups as indicated. *P < 0.05 compared with Sham kidneys (*n* = 3–7); ^#^*P* < 0.05 compared with UUO kidneys with vehicle treatment (*n* = 7).

Previous studies reported that quercetin may inhibit cultured fibroblast activation and ameliorates organ fibrosis in liver, lung and skin^12^,^16^,^17^,^30^. To investigate the anti-fibrotic role of quercetin in the kidneys with obstructive nephropathy, we created a mice model with kidney fibrosis by unilateral ureter obstruction (UUO) in male CD1 mice, and quercetin was administered at 25 mg/kg.day intraperitoneally from 2 days before the operation. The mice were sacrificed and the kidneys were harvested at 2 weeks after UUO surgery. As shown in Fig. 1A,B, periodic acid–Schiff (PAS), Sirius red and Masson staining show that renal fibrotic lesions were significantly ameliorated and interstitial matrix production was decreased in quercetin-treated group at day 14 after UUO, compared with UUO control. Total collagen content in the kidneys was quantified. As shown in Fig. 1C, UUO caused a dramatic induction of total collagen deposition in the kidneys at 2 weeks after surgery, whereas quercetin significantly suppressed collagen deposition. In addition, we also detected the body weight, serum albumin, ALT, AST, total bilirubin (TB) and direct bilirubin (DB) within the mice treated with vehicle or quercetin at 2 weeks after UUO surgery. There was no obvious difference as to those parameters within two groups, suggesting that quercetin treatment for 2 weeks didn’t induce obvious toxicity in the mice (Suppl. Fig. 1).

**Figure 2:**
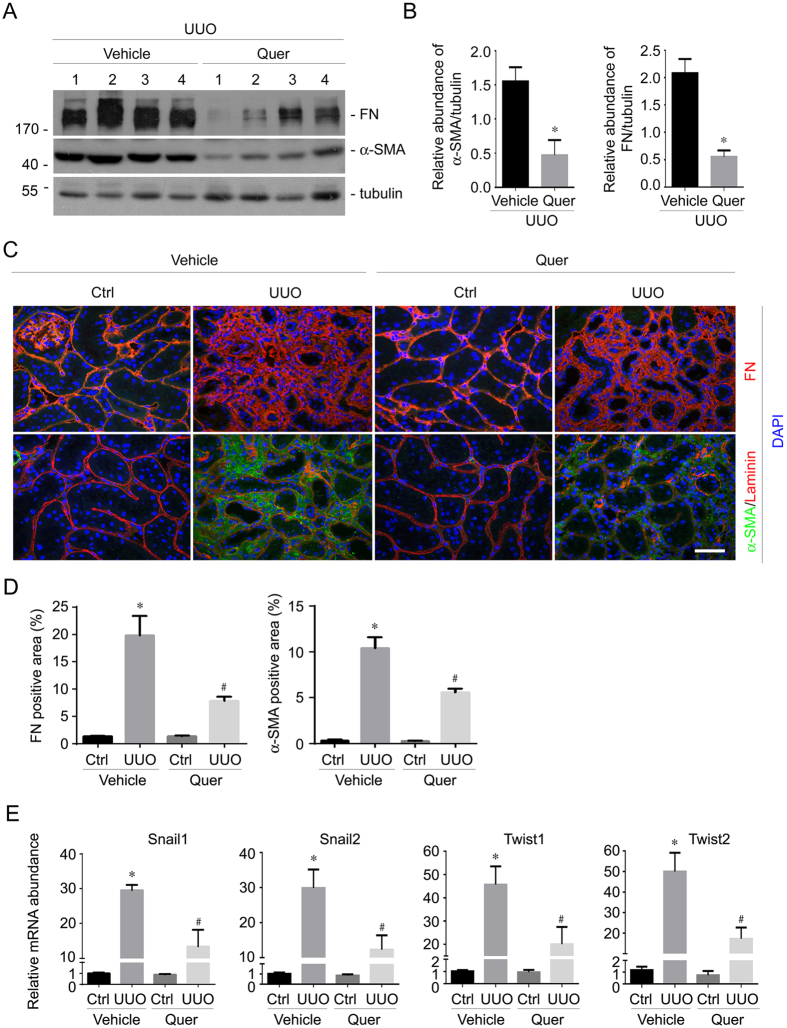
Quercetin reduces FN and α-SMA expression in the UUO kidneys. (**A**) Western blot analyses showing the abundance of FN and α-SMA in the kidneys at 2 weeks after UUO w/o quercetin treatment. The gels were run under the same experimental conditions. (**B**) Graphic presentation showing FN and α-SMA protein abundance from (**A**) in the UUO kidneys. *P < 0.05 compared with UUO kidneys (*n* = 4). (**C**) Representative micrographs showing immuno-staining for FN and α-SMA expression in various groups as indicated. Scale bar = 50 μm. (**D**) Graphic presentation showing the quantitative results for FN and α-SMA immuno-staining in kidney tissue within groups. *P < 0.05 compared with contra lateral kidneys (*n* = 4), ^#^*P* < 0.05 compared with UUO kidneys (*n* = 4). (**E**) Real time PCR analysis for Snail1, Snail2, Twist1 and Twist2 mRNA expression in the kidney tissue among groups. *P < 0.05 compared with contra lateral kidneys (*n* = 5), ^#^*P* < 0.05 compared with UUO kidneys (*n* = 5).

We also detected α-SMA and fibronectin (FN) expression by Western blot analysis. As shown in Fig. 2A,B, FN and α-SMA protein abundance were largely induced on day 14 in mice after UUO. Quercetin could markedly downregulate their expression in the UUO kidneys. Immunofluorescent staining were carried out on frozen kidney specimens that were obtained on day 14 after UUO, and the staining results were similar to Western blotting analyses (Fig. 2C,D).

Snail and Twist are the key transcription factors that regulate the epithelial-mesynchymal transition (EMT) program^31^. To test the role for quercetin in ameliorating tubular cell EMT in the kidneys with UUO nephropathy, we detected the mRNA expression for Twist1, Twist2, Snail1 and Snail2 in the kidneys at 2 weeks after UUO surgery. As shown in Fig. 2E, mRNA expression for these four genes was largely induced in kidneys with UUO nephropathy. Quercetin treatment could not affect the mRNA expression of these four transcription factors in the contra lateral kidneys, whereas in the UUO kidneys, their expression was significantly reduced compared to those in the UUO kidneys treated with vehicle.

### Quercetin diminishes macrophage accumulation and inflammatory cytokines expression in the obstructive kidneys

**Figure 3:**
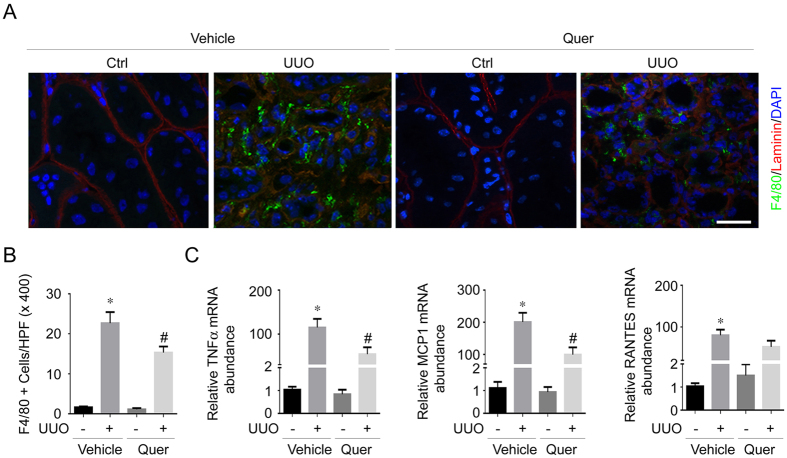
Quercetin diminishes macrophage accumulation and inflammatory cytokines expression in the UUO kidneys. (**A**) Representative micrographs showing the immuno-staining for F4/80 among different groups as indicated. Slides were co-stained with Abs against F4/80 and Laminin. Kidney sections were stained with DAPI to visualize the cell nuclei. Scale bar = 50 μm. (**B**) Graphic presentation showing F4/80 positive macrophage accumulation in the kidneys from various groups. *P < 0.05 compared with contra lateral kidneys (*n* = 7); ^#^*P* < 0.05 compared with UUO kidneys with vehicle treatment (*n* = 7). (**C**) Graphic presentation showing the real-time PCR analysis results for TNFα, MCP1 and RANTES in the kidney tissue among groups. *P < 0.05 compared with contra lateral kidneys (*n* = 5); ^#^*P* < 0.05 compared with UUO kidneys with vehicle treatment (*n* = 5).

We next examined the effects of quercetin on macrophage accumulation and inflammatory cytokine expression in the UUO kidneys. Macrophage was identified by anti-F4/80 immunofluorescent staining. In UUO kidneys, F4/80 positive cell number was largely increased compared with their contra lateral kidneys. Quercetin effectively diminished the accumulation of F4/80-positive inflammatory cells in the UUO kidneys (Fig. 3A,B). We also determined the mRNA abundance of several proinflammatory cytokines including TNFα, monocyte chemotactic protein-1 (MCP1) and the regulated on activation normal T cell expressed and secreted (RANTES) in kidney tissues by real time PCR assay. As shown in Fig. 3C, on day 14 after UUO, TNFα, MCP1 and RANTES mRNA abundance were markedly up-regulated. Administration of quercetin could significantly inhibit TNFα and MCP1 mRNA expression, whereas RANTES mRNA abundance was not altered.

### Quercetin inhibits mTOR and β-catenin signaling activation in the kidneys with obstructive nephropathy

**Figure 4:**
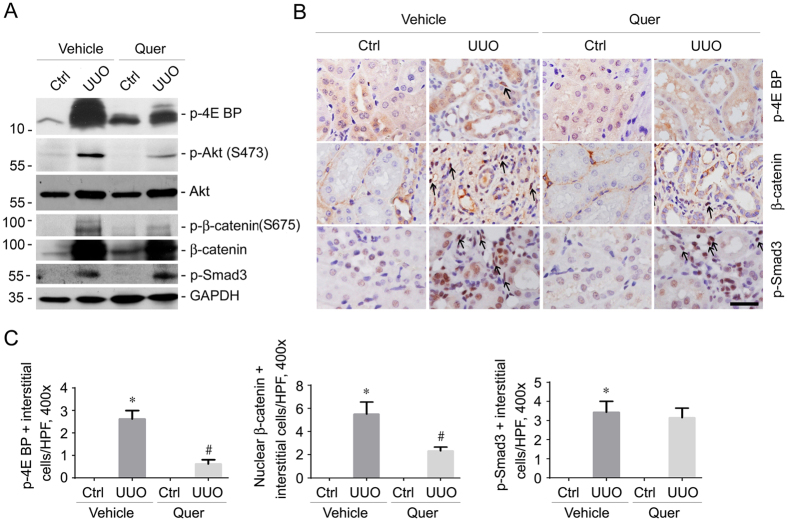
mTOR and β-catenin signaling but not Smad phosphorylation are inhibited by quercetin treatment in the UUO kidneys. (**A**) Western blot analyses showing the abundance of p-4E BP (T37/46), p-Akt (Ser473), p-β-catenin (Ser675) and p-Smad3 (S423/425) in the kidney lysates from mice w/o quercetin treatment. Kidney lysates from each individual animal within each group were pooled together to run the gel. Ab against GAPDH was probed to show the equal loading among different groups. The gels were run under the same experimental conditions. (**B**) Representative micrographs showing the immuno-staining results for p-4E BP (T37/46), β-catenin and p-Smad3 (S423/425) in the kidneys at 2 weeks after UUO. Arrows indicate immuno-staining positive interstitial cells in the kidneys. Scale bar = 20 μm. (**C**) The graphs showing the number for p-4E BP, nuclear β-catenin, or p-Smad3 staining positive interstitial cells within groups. *P < 0.05 compared with contra lateral kidneys (*n* = 4); ^#^*P* < 0.05 compared with UUO kidneys with vehicle treatment (*n* = 4).

Transforming growth factor-β1 (TGFβ1) may activate a panel of intracellular signaling pathways including Smad, β-catenin and mTOR^1^,^18^,^19^,^20^,^21^, and inhibiting such signaling pathways is protective against fibroblast activation and kidney fibrosis. It has been reported that quercetin may inhibit mTOR and β-catenin signaling in cancer cells^26^,^27^,^28^,^29^. We determined these signaling pathways in the kidneys with UUO nephropathy. As shown in Fig. 4A, on day 14 after UUO, p-4E BP (T37/46), p-β-catenin (Ser675), p-Akt (Ser473) and p-Smad3 (S423/425) were all induced in the kidney tissues compared to the contra lateral kidneys, and quercetin markedly decreased the abundance for p-4E BP (T37/46), p-β-catenin (Ser675) and p-Akt (Ser473). It is of note that in the contra lateral kidneys from mice treated with quercetin, p-4E BP was increased compared to those from the vehicle control due to unknown reason. Unexpectedly, p-Smad3 (S423/425) abundance in the UUO kidneys was not changed after quercetin treatment (Fig. 4A). Immunohistochemical staining confirmed the results of Western blotting analyses (Fig. 4B,C).

### Quercetin abrogates kidney fibroblast activation stimulated by TGFβ1 treatment

**Figure 5:**
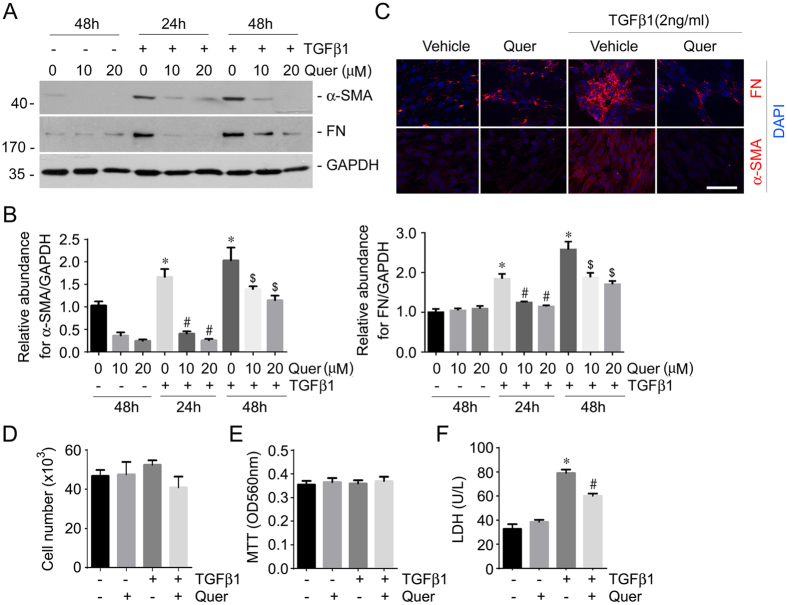
Quercetin diminishes TGFβ1-induced NRK-49F cell activation. NRK-49F cells were pretreated w/o quercetin (10 or 20 μM) and followed by TGFβ1 treatment for 24 or 48 hours. (**A**) Western blot analyses revealing quercetin could downregulate FN and α-SMA expression at both dose and time-dependent manner. The gels were run under the same experimental conditions. (**B**) Graphs showing the quantitative analysis for FN and α-SMA protein expression. *P < 0.05 compared with control cells (*n* = 3); ^#^*P* < 0.05 compared with cells treated with TGFβ1 for 24 hours (*n* = 3); ^$^*P* < 0.05 compared with cells treated with TGFβ1 for 48 hours (*n* = 3). (**C**) Representative micrographs of immunofluorescent staining showing quercetin could suppress TGFβ1-induced FN and α-SMA expression in NRK-49F cells. Scale bar = 20 μm. (**D**) The graph showing the cell number at 48 hours after TGFβ1 treatment within groups as indicated. (*n* = 3). (**E**) The graph showing the result of MTT assay at 48 hours after TGFβ1 treatment within groups. (*n* = 6). (**F**) The graph showing the LDH abundance in the cultured medium at 48 hours after TGFβ1 treatment within groups. *P < 0.05 compared with normal control (*n* = 3); ^#^*P* < 0.05 compared with cells treated with TGFβ1 alone (*n* = 3).

The above data demonstrated that quercetin could inhibit kidney fibrosis in mice with UUO nephropathy. To further investigate the role of quercetin in kidney fibroblast activation, NRK-49F cells, a type of rat kidney interstitial fibroblast cell line, were pretreated with quercetin at 20 μM for 30 mins, followed by TGFβ1 treatment at 2 ng/ml for 24 or 48 hours. TGFβ1 could induce α-SMA and FN expression at both 24 and 48 hours, while pretreatment of quercetin dramatically suppressed TGFβ1-induced α-SMA and FN expression at a dose-dependent manner (Fig. 5A,B). These results were confirmed by immunofluorescent staining for α-SMA and FN in NRK-49F cells (Fig. 5C). Together, these results indicate that quercetin may elicit its anti-fibrotic action by suppressing fibroblast activation initiated by TGFβ1 treatment.

We also examined NRK-49F cell proliferation by counting cell number and MTT assay. There was no difference as to the cell number and the MTT result at 48 hours after TGFβ1 treatment compared to the vehicle control, suggesting that TGFβ1 didn’t affect the NRK-49F cell proliferation within the observation period. Furthermore, Quercetin treatment (20 μM) didn’t affect cell proliferation compared to TGFβ1 treated cells (Fig. 5D,E). LDH abundance in the cultural medium was significantly increased at 48 hours after TGFβ1 treatment compared to vehicle control, suggesting that TGFβ1 (2 ng/ml) treatment may lead to NRK-49F cell injury. LDH abundance was significantly decreased in the cells treated with quercetin (20 μM) plus TGFβ1 compared to the cells treated with TGFβ1 alone, suggesting that quercetin may protect against TGFβ1 induced cell damage (Fig. 5F).

### Quercetin inhibits TGFβ1-induced mTOR and β-catenin but not Smad signaling activation in NRK-49F cells

**Figure 6:**
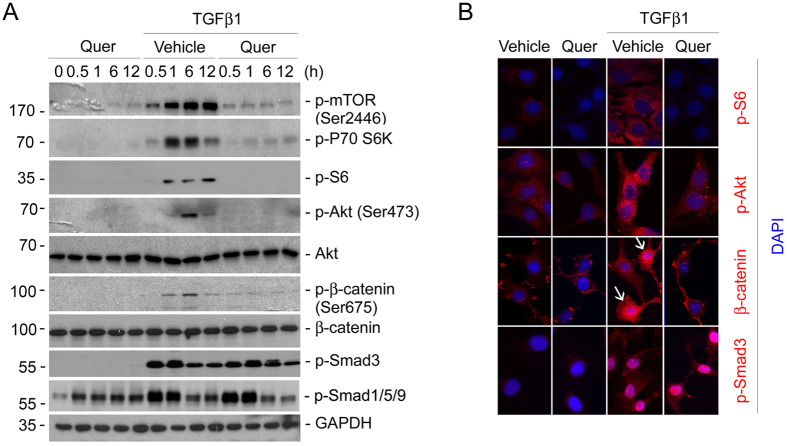
Quercetin inhibits mTOR and β-catenin but not Smad signaling activation stimulated by TGFβ1 in NRK-49F cells. NRK-49F cells were pretreated with quercetin (20 μM) for 30 mins, followed by TGFβ1 (2 ng/ml) treatment and harvested at various time points as indicated. (**A**) Western blot analyses demonstrating that quercetin could reduce the abundance of p-mTOR (Ser2448), p-p70 S6K (T421/S424), p-S6 (S235/236), p-Akt (Ser473), p-β-catenin (Ser675) except p-Smad3 (Ser423/425) and p-Smad1/5/9. The gels were run under the same experimental conditions. (**B**) Representative micrographs of immunofluorescent staining of p-S6 (Ser235/236), p-Akt (Ser473), β-catenin and p-Smad3 (Ser423/425) further confirmed the results of western blotting assay. White arrows indicate the cells with β-catenin nuclear translocation.

It has been reported that quercetin may inhibit mTOR and β-catenin signaling in cancer cells^29^,^32^,^33^,^34^,^35^. Our and the other’s studies demonstrated that both mTOR and β-catenin signaling activation are critical for kidney fibroblast activation and fibrosis^20^,^36^,^37^. To investigate whether quercetin can inhibit these signaling pathways in kidney fibroblasts, NRK-49F cells were treated with quercetin at 20 μM for different time, or pretreated for 30 mins, followed by TGFβ1 treatment at 2 ng/ml for different time as indicated in Fig. 6A. Quercetin treatment alone couldn’t affect mTORC1, mTORC2, β-catenin, or Smad3 signaling activation. Smad1/5/9 phosphorylaton was slightly increased in the cells treated with quercetin. After TGFβ1 treatment, p-mTOR (Ser2448), p-p70 S6K (T421/S424), p-S6 (S234/236), p-4E BP (T37/46), p-Akt (Ser473), p-β-catenin (Ser675), p-Smad3 (S423/425) as well as p-Smad1/5/9 abundance were largely induced at a time-dependent manner. Quercetin could almost completely abolish the phosphorylation of the above molecules except Smad3 and Smad1/5/9 stimulated by TGFβ1. Immunofluorescent staining was also employed to further confirm the results of western blot analysis. As shown in Fig. 6B, NRK-49F cells were harvested at 1 or 3 h after TGFβ1 treatment. Similar to the results of Western blot analysis, phosphorylated S6 and p-Akt (Ser473) were induced and localized in the cytoplasm whereas β-catenin and p-Smad3 underwent nuclear translocation in TGFβ1-treated cells, whereas in the cells pretreated with quercetin, p-S6 (S234/236), p-Akt (Ser473) and β-catenin nuclear translocation were reduced, while Smad3 phosphorylation, a key molecule for canonical TGFβ1 signaling, was not changed. These results indicate that the inhibition of TGFβ1-stimulated mTORC1, mTORC2 and β-catenin but not Smad signaling by quercetin may attribute to its protective role against fibroblast activation and kidney fibrosis.

## Discussion

Here we demonstrated that quercetin may effectively attenuate kidney fibroblast activation and renal interstitial fibrosis via inhibiting mTOR and β-catenin signaling pathways but independent of Smad3 and Smad1/5/9 phosphorylation in kidney fibroblasts.

Quercetin, the most abundant flavonoid ubiquitously contained in vegetables and fruits, has a large spectrum of well characterized biological effects including antioxidative, anti-inflammatory, anticancer, and anti-diabetic activities^38^. Some polyphenols such as quercetin can inhibit cell growth, angiogenesis, and exhibit anti-inflammatory effects in colorectal cancer cell lines^39^. Quercetin may be beneficial in many organs including liver, kidney, skin and bone. It can attenuate symptoms of metabolic syndrome, such as blood pressure, glucose tolerance, and abdominal fat accumulation. Administration of quercetin may suppress the formation of atheromatous plaques with reduced foam cell accumulation, oxidative stress, and inflammatory response in the antherosclerosis mice model^40^. Quercetin can also reduce the levels of oxidative stress and apoptosis in kidney cells in streptozotocin-induced diabetic mice. In addition, quercetin improves hyperuricemia and renal dysfunction via the suppression of renal inflammation in streptozotocin-treated rats^41^. Previous studies reported that quercetin may inhibit lung fibroblast proliferation, diminish extracellular matrix deposition as well as lung fibrosis^14^,^42^. Qin *et al*. tested the *in vitro* anti-fibrotic activities of 21 compounds isolated from plants used in Chinese medicine and methanol extracts of 12 Chinese herbs and revealed that quercetin displays promising potential in preventing kidney fibroblast activation^7^. Jones EA reported that Fas gene expression in the kidneys with UUO nephropathy was significantly inhibited by quercetin treatment in rats. This study may explain the mechanisms for quercetin in protecting against cell apoptosis in kidneys with UUO nephropathy^43^. Additionally, in rats with UUO nephropathy, quercetin can ameliorate kidney structural damage including glomeruli, proximal and distal convoluted tubules (PCT and DCT), Henle’s loop and collecting ducts^44^. Together, these two reports are emphasized on the role and mechanisms of quercetin in ameliorating the damage of nephron and cell apoptosis after UUO. In this study, we demonstrated that quercetin can inhibit kidney fibroblast activation, ameliorate kidney interstitial fibrosis and inflammation in the kidneys with UUO nephropathy. Considering the other reports as to the protective effect for quercetin in UUO rodents, it is highly possible that quercetin may act as a promising anti-fibrotic reagent for patients with chronic kidney diseases.

It is well known that interstitial accumulation of myofibroblasts and macrophages is strongly associated with the progression of renal injury in mice with UUO nephropathy. Quercetin is a potent scavenger of reactive oxygen species including superoxide, and reactive nitrogen species. Moreover, it may also inhibit the release of tryptase, monocyte chemotactic protein-1 (MCP1) and IL-6. In this study, we found that administration of quercetin could largely inhibit myofibroblast accumulation in the kidneys with UUO nephropathy as well as myofibroblast activation in cultured fobroblast cell line, which suggest that kidney interstitial fibroblast should be the direct target of quercetin. Besides the inhibition of fibroblast activation, we found that quercetin could also ameliorate Twist1, Twist2, Snail1 and Snail2 mRNA expression in the kidneys with UUO nephropathy, which suggest that quercetin may also inhibit kidney tubular epithelial cells undergoing EMT in this mouse model with UUO nephropathy. Furthermore, it was reported that quercetin-3-O-glucuronide (Q3GA), a phase II metabolite of quercetin, may be localized in macrophages in human atherosclerotic lesions^45^,^46^. Quercetin significantly attenuates LPS-induced production of tumor necrosis factor-alpha (TNF-α) and interleukin-1beta (IL-1β) in RAW264.7 macrophages. The LPS-stimulated phosphorylation of the inhibitors of kappaB kinase (IKKs), Akt, and c-Jun N-terminal kinase (JNK) are also inhibited by quercetin^47^. In the kidneys with UUO nephropathy, we found that quercetin treatment could diminish macrophage accumulation, TNFα and MCP1 expression. Combined with the other reports, it may be concluded that the anti-fibrotic effect for quercetin in the kidney may be through the inhibition of both interstitial fibroblast activation and inflammation in kidney tissue.

Recently, Nakamura T *et al*. found that methylated quercetin may bind with the transient receptor potential ankyrin 1 (TRPA1) and acts as agonist for this molecule, which may be the receptor for quercetin^48^. Quercetin exerts suppressive effects on the expression of collagen in lung fibroblasts by regulating the translocation of the nuclear factor E2-related factor-2 (Nrf2), an important transcription factor that regulates the expression of HO-1 from the cytoplasm to the nuclei^14^. Quercetin traditionally is considered as a potent antioxidant and anti-inflammatory molecule. However, accumulative evidences found that quercetin can also modulate pathways associated with mitochondrial biogenesis, mitochondrial membrane potential, oxidative respiration and ATP anabolism, and intra-mitochondrial redox status^49^. It is well known that TGFβ1 promotes kidney fibrosis through activating a panel of intracellular signaling pathways. Among of them, Smad signaling is one of the most important in governing TGFβ1-induced fibrosis^22^. However, several other signaling pathways stimulated by TGFβ1 such as mTORC1, mTORC2 and β-catenin are also required for TGF β1-induced fibroblast activation and fibrosis^21^,^23^,^24^,^25^. In glomerular mesangial cells, quercetin may increase Smad7 expression and lead to Smad signaling inactivation^50^. In this study, to elucidate the mechanisms for quercetin in inhibiting kidney fibroblast activation, the alterations of several specific target molecules were examined. We found that in cultured kidney fibroblast cell line, quercetin could largely inhibit TGFβ1-induced mTORC1, mTORC2 and β-catenin activation. Surprisingly, Smad3 phosphorylation was not changed. These observations were also confirmed in the kidneys with UUO nephropathy. Given the critical role for mTOR and β-catenin signaling in promoting fibroblast activation and kidney fibrosis, it may be concluded that inhibition of mTOR and β-catenin but not Smad signaling contribute to the antifibrotic effect of quercetin. More work is needed to clarify the mechanisms for quercetin in inactivating the specific pro-fibrotic signaling pathways and the role for each specific signaling pathway in contribution to the anti-fibrotic effect of quercetin.

In conclusion, our findings decipher the protective effect for quercetin in the kidneys with obstructive nephropathy. In addition, we also found that the anti-fibrotic mechanisms for quercetin may be through targeting β-catenin and mTOR signaling pathways. This study demonstrated that quercetin may represent a novel therapeutic strategy for treatment of kidney fibrosis.

## Methods

### Mice and animal models

Male CD-1 mice weighing approximately 18–20 g were acquired from the specific Pathogen-Free Laboratory Animal Center of Nanjing Medical University and maintained according to the guidelines of the Institutional Animal Care and Use Committee at Nanjing Medical University. UUO was performed as previously reported^20^. The animals were divided into three groups: (1) normal control; (2) UUO mice injected with vehicle (5% DMSO); (3) Mice were treated with quercetin (25 mg/kg.day) from 2 days before UUO surgery to 2 weeks after surgery. Quercetin (cat: Q4951-10 g, Sigma Aldrich, USA) was prepared in a 5% DMSO solution and injected intraperitoneally.

The mice were euthanized on day 14 after UUO. The UUO as well as the contra lateral kidneys were removed. One portion of the kidney was fixed in 10% phosphate-buffered formalin followed by paraffin embedding for histological and immunohistochemical staining. Another portion was immediately frozen in Tissue-Tek optimum cutting temperature compound (Sakura Finetek, Torrance, CA) for cryosection. The remaining kidney tissue was snap-frozen in liquid nitrogen and stored at −80 °C for extraction of RNA and protein. All experiments were performed in accordance with the approved guidelines and regulations by the Animal Experimentation Ethics Committee at Nanjing Medical University. All experimental protocols were approved by the Animal Experimentation Ethics Committee at Nanjing Medical University.

### Cell culture and treatment

Rat kidney interstitial fibroblasts (NRK-49F) cells obtained from American Type Culture Collection (Manassas, VA) were cultured in DMEM/F12 medium supplemented with 10% FBS (Invitrogen, Grand Island, NY). Cells were seeded on six-well culture plates to 60–70% confluence in complete medium containing 10% FBS for 16 hours, and then changed to serum-free medium after washing twice with serum-free medium. Human recombinant TGFβ1 (Cat: 100-B-010-CF, R&D Systems, Minneapolis, MN) was added to the serum-free medium for various periods of time. Quercetin (cat: Q4951-10 g, Sigma Aldrich, USA) dissolved in DMSO was added at 30 mins before TGFβ1 stimulation.

### Histology and immunohistochemistry

Mouse kidney samples were fixed in 10% neutraformaline, embedded in paraffin. 3 μm sections were used for Masson, PAS and Sirius red staining. For immunohistochemical staining, paraffin-embedded kidney sections were deparaffinized, hydrated, antigen-retrieved, and endogenous peroxidase activity was quenched by 3% H_2_O_2_. Sections were then blocked with 10% normal donkey serum, followed by incubation with anti-β-catenin (cat: 61054, BD Biosciences Pharmingen, San Diego, CA), anti-p-4E BP (T37/46) (cat: 2855, Cell Signaling Technology, USA) and anti-p-Smad3 (S423/S425) (cat: ab52903, Abcam, Cambridge, UK) overnight at 4 °C. After incubation with secondary antibody for 1 hour at room temperature (RT), sections were incubated with ABC reagents for 1 hour at room temperature before subjected to substrate 3-amino-9-ethylcarbazole or DAB staining (Vector Laboratories, Burlingame, CA). Slides were viewed with a Nikon Eclipse 80i microscope equipped with a digital camera (DS-Ri1, Nikon, Shanghai, China).

### Immunofluorescent staining

Kidney cryosections at 3 μm thickness were fixed for 15 min in 4% paraformaldehyde, followed by permeabilization with 0.2% Triton X-100 in PBS for 5 min at RT. After blocking with 2% donkey serum for 60 min, the slides were immunostained with primary antibodies against FN, α-SMA, F4/80, and laminin. To visualize the primary antibodies, slides were stained with TRITC or FITC-conjugated secondary antibodies. The percentages for FN or α-SMA staining positive area relative to the total area of each image were measured by Image-Pro Plus (IPP) 6.0 analysis (Media Cybernetics, Silver Spring, USA). Ten images randomly taken from each mouse kidney section were evaluated, and the mean value of the results got from 10 images for each mouse was calculated.

Cells cultured on coverslips were washed twice with cold PBS and fixed with cold methanol/acetone (1:1) for 10 min at −20 °C. After three extensive washings with 1 × PBS, the cells were treated with 0.1% Triton X-100 for 5 min and blocked with 2% normal donkey serum in 1 × PBS buffer for 40 min at RT and incubated with the antibody against p-S6 (S235/236) (cat: 4858, Cell Signaling Technology, USA), β-catenin (cat: 61054, BD Biosciences Pharmingen, San Diego, CA) and p-Smad3 (S423/S425) (cat: ab52903, Abcam, Cambridge, UK), followed by staining with TRITC or FITC-conjugated secondary antibodies. Cells were also stained with 4′, 6-diamidino-2-phenylindole to visualize the nuclei. Slides were viewed with a Nikon Eclipse 80i Epifluorescence microscope equipped with a digital camera.

### Western blot analysis

Cultural NRK-49F cells were lysed in 1 × SDS sample buffer. The kidneys were lysed with RIPA solution containing 1% NP40, 0.1% SDS, 100 mg/ml PMSF, 1% protease inhibitor cocktail, and 1% phosphatase I and II inhibitor cocktail (Sigma Aldrich, St Louis, MO) on ice. The supernatants were collected after centrifugation at 13,000 g at 4 °C for 30 min. Protein concentration was determined by bicinchoninic acid protein assay. An equal amount of protein was loaded into 10% or 15% SDS-PAGE and transferred onto polyvinylidene difluoride membranes. The primary antibodies used as the following: anti-p-mTOR (Ser2448) (cat: 5536, Cell Signaling Technology, USA), anti-p-S6 (S235/236) (cat: 4858, Cell Signaling Technology, USA), anti-p-4E-BP (T37/46), (cat: 2855, Cell Signaling Technology, USA), anti-p-Akt (Ser473), (cat: 3868, Cell Signaling Technology, USA), anti-p-β-catenin (Ser675) (cat: 9567, Cell Signaling Technology, USA), anti-p-Smad1/5/9 (cat: 138201, Cell Signaling Technology, USA), anti-GAPDH mAb (cat: sc25778, Santa Cruz Biotechnology, USA), anti-tubulin (cat: sc53646, Santa Cruz Biotechnology, USA). anti-β-catenin (cat: 61054, BD Biosciences Pharmingen, San Diego, CA), anti-p-Smad3 (S423/S425) (cat: ab52903, Abcam, Cambridge, UK), anti-FN (cat: F3648, Sigma-Aldrich, USA), anti-α-SMA (cat: A5228, Sigma-Aldrich, USA). Quantification was performed by measuring the intensity of the signals with the aid of National Institutes of Health Image software package.

### Semi-quantitative analysis for fibrotic area in the kidney tissue

Kidney sections (3 μm thickness) were stained with Masson-Trichrome kit (cat: HT15-1KT, Sigma Aldrich, USA) according to the manufacturer’s instruction. Accumulated collagen in the interstitial area was stained with aniline blue and showed as light blue under microscope. Ten fields were randomly selected for each kidney section. The percentage of interstitial fibrotic area to the selected field for ten randomly selected fields from each section were analyzed with Image Pro Plus 6.0, and an average percentage of kidney fibrotic area for each section was calculated.

### Quantitative determination of total collagen content in kidney tissue

The kidney tissue collagen content was quantitated. Briefly, 3 μm sections of paraffin-embedded tissue were stained with Sirius red F3BA and Fast Green FCF overnight. After washing three times with 1 × PBS buffer, the dye was eluted from tissue sections with 0.1N sodium hydroxide methanol. Absorptions at 540 nm and 605 nm were determined for Sirius red F3BA and Fast Green FCF binding protein, respectively. This assay provided a simple, relative measurement of the ratio of collagen to total protein, which was expressed as micrograms per milligram of total protein.

### RNA isolation and real-time quantitative RT-PCR

Total RNA was extracted using Trizol reagent (cat: 15596018, Invitrogen, USA) according to the manufacturer’s instruction. cDNA was synthesized using 1 μg of total RNA, ReverTra Ace (cat: R111-02, Vazyme, Nanjing, China), and oligo (dT) 12–18 primers. Gene expression was measured by real-time PCR using real-time PCR Master Mix reagents (cat: Q141-02, Vazyme, Nanjing, China) and 7300 real-time PCR system (Applied Biosystems, Foster City, CA). GAPDH was used as an internal control. For real-time PCR analysis, the relative amount of mRNA or gene to internal control was calculated using the equation 2^ΔCT^, in which ΔCT = CT_gene_ -CT_control._

### Statistical analysis

Data were expressed as mean ± s.e.m. Student’s unpaired t-test for comparison of means was used to compare between two groups. Statistical analysis of data was performed using the GraphPad Prism software (GraphPad Software, California, USA). *P* < 0.05 was considered statistically significant.

## Additional Information

**How to cite this article**: Ren, J. *et al*. Quercetin Inhibits Fibroblast Activation and Kidney Fibrosis Involving the Suppression of Mammalian Target of Rapamycin and β-catenin Signaling. *Sci. Rep*. **6**, 23968; doi: 10.1038/srep23968 (2016).

## Supplementary Material

Supplementary Information
