# Supplementary material for: Quercetin Inhibits Fibroblast Activation and Kidney Fibrosis Involving the Suppression of Mammalian Target of Rapamycin and β-catenin Signaling
Source: Sci Rep. 2016 Apr 7;6:23968. doi: 10.1038/srep23968 (PMC4823739; doi:10.1038/srep23968)
Supplement: Supplementary Information [file srep23968-s1.pdf]

Quercetin Inhibits Fibroblast Activation and Kidney Fibrosis Involving the Suppression of Mammalian Target of Rapamycin and β-catenin Signaling

Jiafa Ren[1], Jianzhong Li[1], Xin Liu[1], Ye Feng[1], Yuan Gui[1], Junwei Yang[1], Weichun He[1] and Chunsun Dai[1,2¶]

[1] Center for Kidney Disease, 2nd Affiliated Hospital, Nanjing Medical University, 262 North Zhongshan Road, Nanjing, Jiangsu, China

[2] State Key Laboratory of Reproductive Medicine, Institute of Toxicology, Nanjing Medical University, Nanjing, Jiangsu, China

[¶]To whom correspondence should be addressed:

Chunsun Dai, MD/PhD, Center for Kidney Diseases, 2nd Affiliated Hospital, Nanjing Medical University, 262 North Zhongshan Road, Nanjing, Jiangsu, P.R. China. 210003 (86-25-52636077, E-mail: daichunsun@njmu.edu.cn to Dai C).

Supplemental Figure 1: The general parameters of the mice at 2 weeks after UUO surgery w/o Quercetin treatment. The mice were treated with vehicle or quercetin (25mg/kg.day, ip). At 2 weeks after UUO surgery, the blood from each mouse was harvested. The body weight and the biochemical parameters were examined. (A) Body weight, (B) Serum albumin level, (C) ALT; (D) AST; (E) TB, Total bilirubin; (F) DB, Direct bilirubin. (*n*=4).

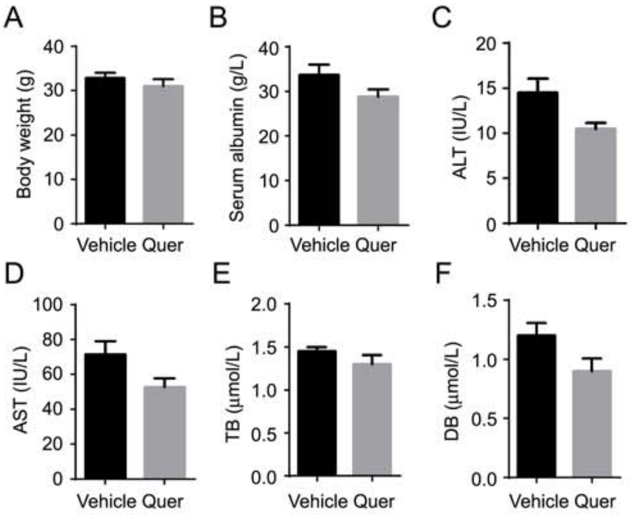

A
Body weight (g)
40
30
20
10
0
Vehicle Quer
B
Serum albumin (g/L)
40
30
20
10
0
Vehicle Quer
C
ALT (IU/L)
20
15
10
5
0
Vehicle Quer
D
AST (IU/L)
100
80
60
40
20
0
Vehicle Quer
E
TB (μmol/L)
2.0
1.5
1.0
0.5
0.0
Vehicle Quer
F
DB (μmol/L)
1.5
1.0
0.5
0.0
Vehicle Quer
